# Analysis of Baseline Computerized Neurocognitive Testing Results among 5–11-Year-Old Male and Female Children Playing Sports in Recreational Leagues in Florida

**DOI:** 10.3390/ijerph14091028

**Published:** 2017-09-07

**Authors:** Karen D. Liller, Barbara Morris, Jessica Fillion, Yingwei Yang, Omonigho M. Bubu

**Affiliations:** 1 College of Public Health, University of South Florida, Tampa, FL 33612, USA; jfillionatc@gmail.com (J.F.); yingweiyang@health.usf.edu (Y.Y.); obubu@health.usf.edu (O.M.B.); 2 Florida Hospital Wesley Chapel, Wesley Chapel, FL 33544, USA; barbara.morris@ahss.org

**Keywords:** concussions, neurocognitive testing, children’s injuries, sports injuries, recreational leagues

## Abstract

There is a paucity of data related to sports injuries, concussions, and computerized neurocognitive testing (CNT) among very young athletes playing sports in recreational settings. The purpose of this study was to report baseline CNT results among male and female children, ages 5–11, playing sports in Hillsborough County, Florida using ImPACT Pediatric, which is specifically designed for this population. Data were collected from 2016 to 2017. The results show that 657 baseline tests were conducted and t-tests and linear regression were used to assess mean significant differences in composite scores with sex and age. Results showed that females scored better on visual memory and in general as age increased, baseline scores improved. The results can be used to build further studies on the use of CNT in recreational settings and their role in concussion treatment, management, and interventions.

## 1. Introduction

### 1.1. Concussion Injuries in Young Children

Much of the literature on sports concussion injuries has been directed towards high school, collegiate, and professional athletes [1,2]. There are very limited data on the incidence of sports-related concussions among pre-high-school-age youth and among those playing in youth clubs and in recreational sports [3]. A recent systematic review and meta-analysis did show for males and females 18 years of age and younger that the overall pooled concussion rate was estimated at 0.23 per 1000 athletic exposures, with the highest incidence rates in rugby, hockey, and American football [4]. However, the majority of studies did not report age, gender-specific incidence rates, or an operational definition of concussion [4].

For the youngest athletes, many sports or recreational activities are informal and not organized for reporting injuries, so both incidence and recovery data for this group are based upon estimates [5]. However, concussions among younger children and adolescents continue to be a serious issue. The Centers for Disease Control and Prevention reported that the number of sports and recreation emergency department visits for traumatic brain injury (TBI) among persons ≤19 years of age increased 62% and that the rate of TBI visits increased 57% from 2001 to 2009 [6].

### 1.2. Computerized Neurocognitive Testing (CNT) for Concussions of Young Children and Adolescents

Computerized neurocognitive testing (CNT) is done for baseline and follow-up concussions among athletes. Certified athletic trainers (ATCs) can provide this testing and are typically the first providers to identify and evaluate injured players [7]. They are also critical in providing concussion management and return-to-play decisions [7]. Age appropriate concussion-assessment tools should be used in younger athletes [7]. Most ATCs (93%) use the computerized test ImPACT (Immediate Post Concussion Assessment and Cognitive Testing). A recent systematic review of concussion management in children and adults concluded that both computerized and standard paper and pencil neuropsychological tests were able to detect cognitive impairments within 48 h of injury and in the short/medium time period (10–14 days) post-injury [8]. However, the authors could not recommend their widespread use due to lack of research, especially verifying the psychometric properties of the most common tools, particularly in young athletes. The authors report that CNT is a tool at the disposal of the clinician and should not be a stand-alone test [8]. Research is also needed to determine the validated role of CNT in prevention strategies [8].

Differences in sex have been shown to affect CNT results. Studies using the ImPACT test have shown female athletes in middle school, high school, and college exhibited a statistically significant greater aggregate symptom severity and longer reaction time than males [9,10,11] at baseline. A study conducted by Cottle and colleagues showed that female collegiate students performed better on ImPACT’s visual memory, visual motor speed, and reaction time than males [12]. However, no studies were found assessing sex differences using the new tool, ImPACT Pediatric, among 5–11-year-old children.

To determine if ImPACT Pediatric can be successfully used with children playing sports in recreational settings and to have baseline scores in case of future concussions in this population, we collected baseline ImPACT Pediatric neurocognitive results on 5–11-year-old male and female children playing sports in recreational leagues in Hillsborough County, Florida. We also wished to determine if the baseline findings differed for this population due to sex and age.

## 2. Materials and Methods

This study of neurocognitive results is part of a larger study focused on injuries among 5–11-year-old children who play sports in recreational leagues in Hillsborough County, Florida. One lead ATC collects the sports injury data on athletes and 13 additional ATCs assist when needed with baseline neurocognitive testing. The selection of leagues and venues (field sites) to participate in this research was determined by the number and representation of athletes and the number of ATCs that could be hired. ATCs were trained in the use of the ImPACT tool and working relationships were established with the athletic complex, athletic directors, and coaches before the start of the study. An internal advisory group comprised of practitioners, researchers, and a neuropsychologist were available for review and discussion of findings. The participant pool for this study was drawn from the 1511 children who consistently played baseball, softball, soccer, and American football in the setting used for this research.

Baseline neurocognitive data were collected with ImPACT Pediatric (research version) on available and consenting athletes. Children were tested before participating in practices or games to avoid fatigue when responding. This project has been approved by the University of South Florida Institutional Review Board and is funded by the National Operating Committee on Standards for Athletic Equipment.

### 2.1. ImPACT Pediatric

According to information from the most recent Impact Pediatric Administration and Interpretation Manual (contact impacttest.com for availability), ImPACT Pediatric was developed to fill the void of adequate assessment solutions for younger children for concussion testing. It is the only U.S. Food and Drug Administration (FDA)- cleared concussion assessment tool for ages 5–11. This tool, developed for 5–11-year-old children, is tablet-based for ease of use and portability. The principles behind this tool include: tests must be brief and fun, repeatable, sensitive to after-effects of injury, and not rely on reading skills. Test providers must complete the appropriate online training and have a basic level of knowledge regarding the medical assessment of concussion and neurocognitive factors. Post-injury testing must be done by properly trained and licensed healthcare professionals with specific knowledge and experience in interpreting neurocognitive test results. Administration time is between 15 and 20 min. The test is to be administered by professionals specifically trained, including psychologists, ATCs, and nurses. Reliability testing to date has shown that test-retest-correlation scores are high overall with intraclass correlations ranging from 0.34–0.825, with the large majority of values in the 0.7 and 0.8 range, including word lists, design rotation, stop and go, memory touch, and picture match. ImPACT Pediatric was standardized with a sample of 5–11-year-old children from 2012 to 2015 in several states, including Georgia, Maryland, Michigan, New York, New Jersey, Pennsylvania, Texas, and Virginia. The ImPACT Pediatric manual has shown the test has good validity, including face and concurrent validity.

An exploratory factor analysis with varimax rotation using Eigenvalues >1.0 for factor extraction was conducted with ImPACT Pediatric. Results showed a four-factor solution of word memory (word items), sequencing/attention (memory touch), visual memory (picture match average taps and average time), and reaction time (stop and go average time and design rotation number correct).

### 2.2. Statistical Analysis

Descriptive statistics were calculated for sex, age, and sports played. To examine whether ImPACT scores varied by sex, four independent sample *t*-tests were conducted separately using the mean score of each ImPACT summary scores (i.e., sequencing/attention; word memory; visual memory; and reaction time). Our populations were independent and we determined the data were normally distributed using the Shapiro-Wilks test, and examined homogeneity of population variance using the Levene’s *F* Test. Word memory, visual memory, and reaction time had unequal variance and, as such, their results were interpreted using the Satterthwaite adjustment for the degrees of freedom.

To examine the effect of age on the ImPACT scores, a linear regression model was utilized. The model was adjusted for sex, and interaction terms for age and sex were examined. Statistical significance for all tests conducted was determined using Bonferroni correction (*p* ≤ 0.0125) since we conducted four separate independent *t*-tests and four separate linear regressions, one for each ImPACT Pediatric composite score.

## 3. Results

Study participants included six hundred and fifty-seven (657) 5–11-year-old children (mean age ± SD: 8.31 ± 1.62) who play sports in recreational leagues in Hillsborough County, Florida. Fifty percent of children tested played soccer and the least number of children played American football (10%). Of the 657 participants, 431 were males (66%), and 226 were females (34%). Recreational sports played were American football (*N* = 65), soccer (boys *N* = 201; girls *N* = 128), baseball (*N* = 168), and softball (*N* = 95). See Table 1 for further demographic information.

Results from the independent *t*-tests showed a significant difference in visual memory scores across sex, with females performing better, as shown by the less number of taps and faster average time to respond. Other summary scores did not show significant differences between males and females. See Table 2.

Results from the adjusted linear models showed significant effects of age on three of the neurocognitive domains including sequencing/attention, word memory, and visual memory. No significant age effects were seen with reaction time. Generally, participants performed better with increasing age (See Table 3). It is important to note that higher scores in visual memory correspond to lower skills in this area, as this composite is comprised as the number of taps (which shows memory of items) and the average time to complete the task. There were no significant interactions between age and sex.

## 4. Discussion

Our findings showed that females scored better on visual memory than males, however, there were no other significant differences in sex for the other neurocognitive domains. We did find that as children age and mature, their baseline scores did generally improve as might be expected with maturity. This is why it is important for athletes to be assessed for baseline findings on a regular basis so that any post-concussion scores can be compared to comparable baseline scores. Our results related to sex are somewhat congruent with data from older athletes noting variations in neurocognitive performance between sexes [13,14]. However, older athletes are tested with ImPACT and not ImPACT Pediatric. Covassin et al., in a study examining performance on neurocognitive tests in college athletes, showed that females performed better than males on verbal memory, but males performed better on visual memory [14]. In our study, it was the reverse, with females performing better for visual memory. We did not demonstrate variations in the other neurocognitive domains. However, studies in college athletes showed differences in visual motor speed and reaction time, with females performing better [15].

The role of CNT in regard to concussion detection and management is still developing for young athletes. When using CNT for concussions, some authors have recommended the use of age-matched norms in the absence of baseline scores [16]. However, children who suffer from a sports-related concussion may not be representative of general norms. They may be mostly male, and have attention-deficit/hyperactivity disorder (ADHD) or other learning disorders which can falsely lower test scores on domains such as attention, memory, and learning. That is why the collection of individualized preinjury scores (baseline scores) is recommended [8]. There also is debate about when CNT should be done with pediatric populations. Some practitioners recommend testing only when the athlete is asymptomatic, others have used testing in the acute symptomatic stages to monitor recovery and delayed recovery. While there are challenges with neurocognitive testing, this information can still be important in reference to the child’s ability to retain new information, process verbal information, and focus attention [16].

The results of our study showed that we were able to successfully collect baseline CNT data in our study population playing sports in recreational settings. This was accomplished by working with the athletic complex before the study to enlist support and having trained ATCs available to administer the CNT. We will continue to collect data for another year. There is need for further study and determination of the role of CNT testing related to outcome data. Once a better understanding for the role of and impact of concussions on very young athletes becomes known, the contributions of CNT baseline and follow-up testing may be better assessed. CNT tools providing information about concussions and their severity and outcome may help with the development of multifactorial interventions planned by a multidisciplinary team that includes the athlete, medical community, schools, coaches, and parents. It may be that a one-size-fits all approach will not be successful with young athletes of different sexes and varying ages. However, education is vital so that all individuals understand the signs and symptoms of concussion and respond appropriately with follow-up treatment and care [16]. We agree with researchers’ recommendations that when at all possible these interventions should focus on a socioecological approach, utilizing individual, interpersonal, community, and society and policy factors [17].

Limitations of this research include lack of generalizability due to data being collected at one sports complex in Florida. Also, the testing of the athletes may have produced varied results, however, the ATCs were thoroughly trained on ImPACT Pediatric and were observed on two or more testing sessions by the study researchers for consistency. The guidelines for use of the instrument states that ATCs are a group of healthcare professionals who can administer the tool. We also had access to an advisory committee that included a neuropsychologist if discrepancies or difficulties in testing occurred—none of which did. Also, the advisory committee had access to all of our study findings. Finally, while the baseline results did show improvement with age, what is important is that baseline scores are continually assessed on the study population so that proper individual comparisons can be made as the children mature.

## 5. Conclusions

The results of this study show that ImPACT Pediatric can be successfully administered in recreational settings through the use of qualified healthcare professionals (ATCs). We also showed that baseline ImPACT Pediatric scores showed no significant differences due to sex except for visual memory. However, scores generally did significantly improve with age. Continued use of this tool and monitoring of concussions for this population will be important to do for comparison of post-concussion findings to baseline results and for future research and program development.

## Figures and Tables

**Table 1:** Participant Demographics, *N* = 657.

Variables	*N* (%)	Age (Mean ± SD)
**Total**	657 (100%)	8.31 ± 1.62
**Sex**		
Male	431 (66%)	8.29 ± 1.47
Female	226 (34%)	8.33 ± 1.70
**Age**		
5-year-old	28 (4%)	
6-year-old	63 (10%)	
7-year-old	125 (19%)	
8-year-old	146 (22%)	
9-year-old	121 (18%)	
10-year-old	102 (16%)	
11-year-old	72 (11%)	
**Sports**		
American Football	65 (10%)	8.51 ± 1.55
Baseball	168 (26%)	7.55 ± 1.71
Softball	95 (14%)	8.09 ± 1.57
Boys Soccer	201 (31%)	8.86 ±1.50
Girls Soccer	128 (19%)	8.46 ± 1.33

**Table 2:** ImPACT Scores by Sex, *N* = 657.

Sex	Sequencing/Attention		Word Memory		Visual Memory		Reaction Time	
	Mean ± SD	*p*-Value	Mean ± SD	*p*-Value	Mean ± SD	*p*-Value	Mean ± SD	*p*-Value
Male (431)	4.94 ± 1.71	0.06	5.54 ± 0.82	0.04	37.36 ± 7.48	0.01 *	5.20 ± 0.57	0.15
Female (226)	4.67 ± 1.59		5.67 ± 0.73		35.93 ± 6.35		5.10 ± 0.89	

* *p* ≤ 0.0125.

**Table 3:** Linear regression for age and sex, *N* = 657.

Composite	Beta Estimate	Standard Error	*t*-Value	*p*-Value
**Sequencing/Attention**				
Age	0.46	0.04	12.57	<0.0001 *
Sex (Ref: Male)				
Female	0.56	1.03	−0.54	0.59
Age x Sex	0.02	0.08	−0.27	0.79
**Word Memory**				
Age	0.2	0.02	11.24	<0.0001 *
Sex (Ref: Male)				
Female	−0.81	0.5	−1.62	0.11
Age x Sex	0.02	0.04	0.55	0.58
**Visual Memory**				
Age	−1.54	0.16	−9.32	<0.0001 *
Sex (Ref: Male)				
Female	−0.71	4.64	−0.15	0.88
Age x Sex	−0.46	0.35	−1.30	0.19
**Reaction Time**				
Age	0.03	0.02	1.49	0.14
Sex (Ref: Male)				
Female	−0.53	0.48	−1.10	0.27
Age x Sex	0.0005	0.04	0.15	0.88

* *p* ≤ 0.0125.

## References

[1] Williams R.M., Welch C.E., Weber M.L., Parsons J.T., Valovich McLeod T.C. (2014). Athletic trainers’ management practices and referral patterns for adolescent athletes after sport-related concussion. Sports Health.

[2] Cournoyer J., Tripp B.L. (2014). Concussion knowledge in high school football players. J. Athl. Train..

[3] Graham R., Rivara F.P., Ford M.A., Spicer C.M., Committee on Sports-Related Concussions in Youth, Board on Children, Youth, and Families, Institute of Medicine, National Research Council (2014). The National Academies Collection: Reports funded by National Institutes of Health. Sports-Related Concussions in Youth: Improving the Science, Changing the Culture.

[4] Pfister T., Pfister K., Hagel B., Ghali W.A., Ronksley P.E. (2016). The incidence of concussion in youth sports: A systematic review and meta-analysis. Br. J. Sports Med..

[5] Conder R.L., Conder A.A. (2015). Sports-related concussions. NC. Med. J..

[6] Centers for Disease Control and Prevention (CDC) (2011). Nonfatal traumatic brain injuries related to sports and recreation activities among persons aged ≤19 Years—United States, 2001–2009. MMWR Morb. Mortal. Wkly. Rep..

[7] Broglio S.P., Cantu R.C., Gioia G.A., Guskiewicz K.M., Kutcher J., Palm M., Valovich McLeod T.C. (2014). National athletic trainers’ association position statement: Management of sport concussion. J. Athl. Train..

[8] Davis G.A., Anderson V., Babl F.E., Gioia G.A., Giza C.C., Meehan W., Moser R.S., Purcell L., Schatz P., Schneider K.J. (2017). What is the difference in concussion management in children as compared with adults? A systematic review. Br. J. Sports Med..

[9] Zuckerman S.L., Apple R.P., Odom M.J., Lee Y.M., Solomon G.S., Sills A.K. (2014). Effect of sex on symptoms and return to baseline in sport-related concussion. J. Neurosurg. Pediatr..

[10] Ono K.E., Burns T.G., Bearden D.J., McManus S.M., King H., Reisner A. (2016). Sex-based differences as a predictor of recovery trajectories in young athletes after a sports-related concussion. Am. J. Sports Med..

[11] Mormile M.E., Hunt T.N. (2016). The role of gender in neuropsychological assessment in healthy adolescents. J. Sport Rehabil..

[12] Cottle J.E., Hall E.E., Patel K., Barnes K.P., Ketcham C.J. (2017). Concussion baseline testing: Preexisting factors, symptoms, and neurocognitive performance. J. Athl. Train..

[13] Covassin T., Elbin R.J., Larson E., Kontos A.P. (2012). Sex and age differences in depression and baseline sport-related concussion neurocognitive performance and symptoms. Clin. J. Sport Med..

[14] Covassin T., Swanik C.B., Sachs M., Kendrick Z., Schatz P., Zillmer E., Kaminaris C. (2006). Sex differences in baseline neuropsychological function and concussion symptoms of collegiate athletes. Br. J. Sports Med..

[15] Covassin T., Elbin R., Kontos A., Larson E. (2010). Investigating baseline neurocognitive performance between male and female athletes with a history of multiple concussion. J. Neurol. Neurosurg. Psychiatry.

[16] Guskiewicz K.M., Valovich McLeod T.C. (2011). Pediatric sports-related concussion. PM R.

[17] Register-Mihalik J., Baugh C., Kroshus E., Zachary Y.K., Valovich McLeod T.C. (2017). A multifactorial approach to sport-related concussion prevention and education: Application of the socioecological framework. J. Athl. Train..

